# Centenarians exposed to the Spanish flu in their early life better survived to COVID-19

**DOI:** 10.18632/aging.203577

**Published:** 2021-09-27

**Authors:** Michel Poulain, Dany Chambre, Giovanni Mario Pes

**Affiliations:** 1 IACCHOS Université Catholique de Louvain, Louvain-la-Neuve, Belgium; 2 Estonian Institute for Population Studies, Tallinn University, Tallinn, Estonia; 3 Independent Gerontologist, Estaimpuis, Belgium; 4 Department of Medical, Surgical and Experimental Sciences, University of Sassari, Sassari, Italy

**Keywords:** COVID-19, Spanish flu, centenarians, oldest-old

## Abstract

Background: Although it is known that mortality due to COVID-19 increases progressively with age, the probability of dying from this serious infection among the oldest-old population is little known, and controversial data are found in literature.

Methods: We examine the mortality by year and month of birth of Belgians who had turned 100 during the current COVID-19 pandemic and whose birth fell on the years around the end the First World War and the outbreak of the H1N1 "Spanish flu" pandemic.

Findings: The COVID-19 mortality of the "older" centenarians is significantly lower than that of "younger" centenarians, and this difference between the two groups reaches a maximum on August 1, 1918 as the discriminating cut-off date of birth. Having excluded the plausible impact of the end of WWI it becomes clear that this date corresponds to the time of reporting the first victims of the Spanish flu pandemic in Belgium.

Interpretation: In this study, the striking temporal coincidence between the outbreak of the Spanish flu epidemic and the birth of the cohorts characterized by greater fragility towards COVID-19 in 2020 strongly suggests a link between exposure to 1918 H1N1 pandemic influenza and resistance towards 2020 SARS-Cov-2. It can be speculated that the lifetime persistence of cross-reactive immune mechanisms has enabled centenarians exposed to the Spanish flu to overcome the threat of COVID-19 a century later.

## INTRODUCTION

Globally, the COVID-19 pandemic has become one of the most serious challenges to public health, affecting millions of lives and families [1]. With 11,500,000 people and 20,000 deaths attributed to COVID-19 in 2020, Belgium exhibited the highest mortality rate per inhabitant worldwide [2]. The first case of infection by the coronavirus was confirmed on February 2 and the first death attributed to the virus was reported on March 10. Death waves occurred twice in 2020, which peaked in April and November [3].

Among the 127,407 deaths recorded in 2020, 56,258 were of people aged 85 and above, constituting a 35.7% increase compared with the average number of deaths from 2009 to 2019, while the excess mortality was 18.3% for the whole population. A total of 1079 centenarians died in 2020 compared with an average of 826 in the period 2009-2019, representing a 30.6% increase. In 2020, 929 centenarians died starting from March 10 when the first death due to COVID-19 was recorded. When data on overall mortality among centenarians by month is compared with the deaths of 190 centenarians ascribable to COVID-19 [4], it clearly appears that the excess mortality risk (EMR, hereafter) to centenarians is related to COVID-19 excluding some additional deaths due to the heat wave in early August.

In order to explain the disproportionate increase in the number of deaths among the oldest old, compared with that usually observed during the last decade, a more in-depth analysis is required, focusing on the size of birth cohorts and, more precisely, on the distribution, by year of birth, of the number of people alive in Belgium on March 10, 2020. Interestingly, at the end of World War I (WWI), the number of births varied largely and almost doubled starting from August 1919 (9 months after the end of WWI). Consequently, an increased number of neo-centenarians emerged in Belgium starting from the summer of 2019; 493 among the 929 centenarians who died from March 10 were between their 100^th^ and 101^st^ birthdays, that is, 53% compared with 39% in 2019.

### Centenarians and COVID-19 pandemic

Although widely emphasized by the media [5], the resilience of centenarians to COVID-19 remains a controversial issue that is now increasingly attracting researchers. Couderc et al. [6], in a study on 321 nursing home residents in Southern France, including 12 centenarians, reported a higher mortality rate among these centenarians (50% vs 24.6%) compared with other younger residents, corresponding to one of the lowest survival rates compared with other published series [7]. Similarly, Marcon et al. [8], in a study on 42 centenarians from North-Eastern Italy as part of the CaT (Centenari a Trieste) project, observed that COVID-19-related mortality among long-lived individuals was higher than that of the population between 50 and 80 years of age, and that the mortality rate among the oldest women exceeded that of men. Overall, these data suggest that despite their ability to reach the extremes of human lifespan, centenarians are not particularly resistant to COVID-19 compared with the general population. In this context, a first research question arises: Does the oldest old, and more specifically the centenarians, die more than usually during the COVID-19 pandemic?

## MATERIALS AND METHODS

The following data is used in this analysis:

- Number of deaths by single year of age at death recorded in Belgium in 2009-2020 [9] and more detailed data for the years 2016-2020 with dates of birth and death and population stock provided the Centre de Démographie (Uclouvain).
- Number of deaths of centenarians attributed to COVID-19 provided by Sciensano by year of birth, age at death, and month of death in 2020. Registered COVID-19 related deaths include confirmed and possible COVID-19 deaths. A case can be confirmed either by a chest CT scan with clinical presentation or a laboratory test. Possible deaths are those who meet the clinical criteria, whether or not there is an epidemiological link to a confirmed case [3].

In this study, mortality risk is defined as the probability of a given birth cohort of centenarians alive at the beginning of the period to die between March 10 and December 31, 2020. Notably, this is not the probability to die between two exact ages but between two exact dates.

The EMR is evaluated by comparing these observed probabilities with the expected ones. The latter are linearly extrapolated from the corresponding observed probability for the years 1991-2019 and smoothed for the oldest ages. A special attention is devoted to estimating the probability of death between March 10^th^ and December 31^st^, excluding the mortality at the beginning of the year. Since the number of centenarians alive on March 10^th^ for each single month and year of birth was small, a moving average with a bandwidth of 12 months was used for calculating these probabilities. The EMR is equivalent to the ratio between the observed and expected number of deaths for the period of the pandemic. To compute the corresponding confidence interval the usual formulas for mortality ratio is used.

In the present study two questions are addressed that are not necessarily related to each other. The first is whether the resistance of centenarians to SARS-Cov-2 infection is generally greater than that of younger individuals. Despite the strong theoretical interest of this question, the literature on this subject is scanty and does not provide sufficient elements to draw a definitive conclusion. The second and more specific question is whether the mortality of centenarians during the COVID-19 epidemic may depend on whether they were born before or after the 1918 flu pandemic. Unlike the first question, which could in principle be answered using available data, responding to the second is much more problematic, given the extremely small number of individuals exposed to both epidemics.

## RESULTS

**Figure 1:**
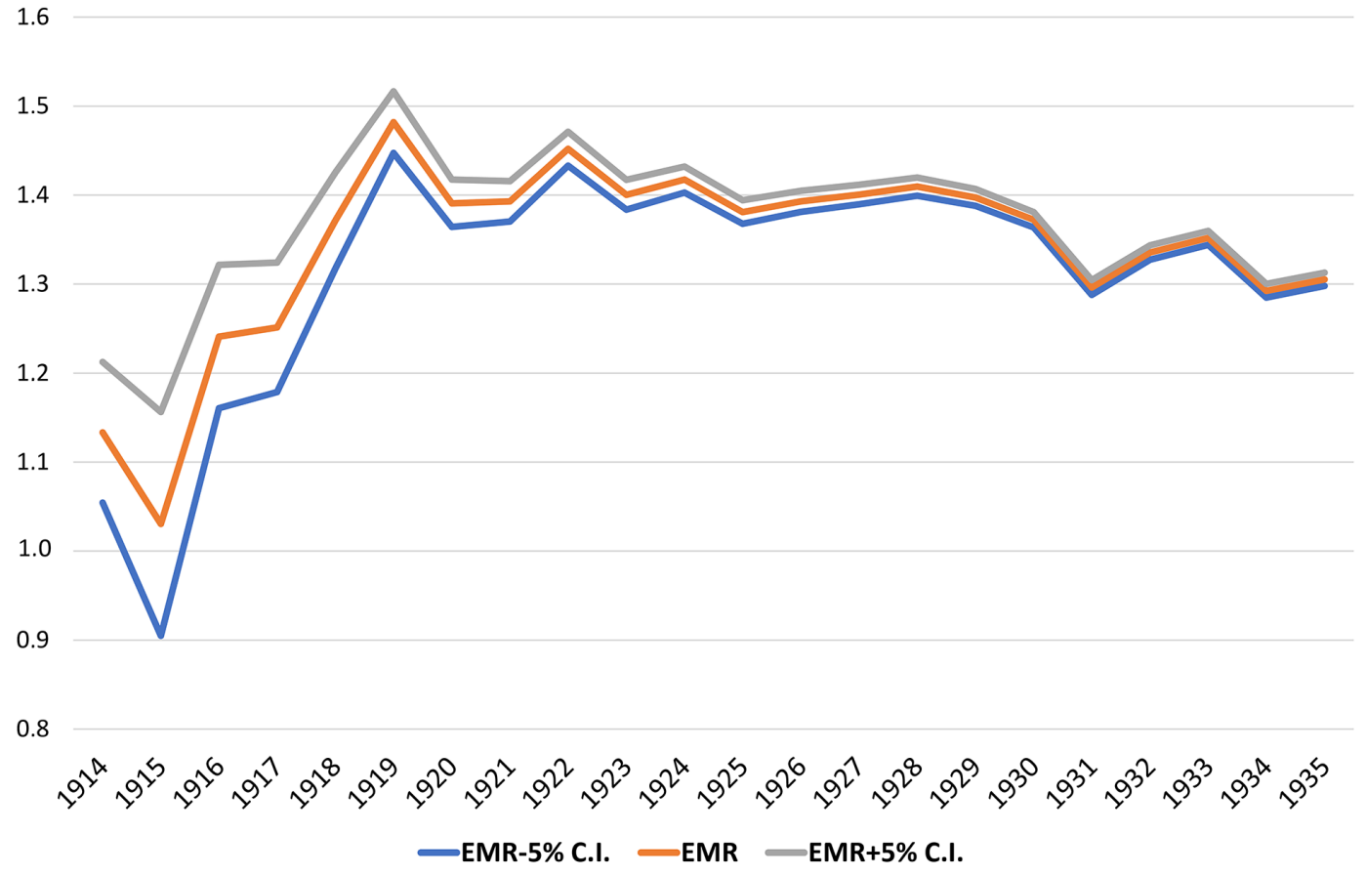
EMR during the pandemic by single year of birth from 1914 to 1935 with a 5% confidence interval.

Figure 1 illustrates the EMR calculated by single year of birth during the pandemic for people born till 1935 (see detailed data in Supplementary Table 1).

The EMR by single year of age is considerably stable except for centenarians born in 1919 or earlier; the EMR suddenly drops to virtually approach unity for those born in 1915. Hence, a second research question, complementary to the previous one, can be raised: Is there a significant survival difference concerning the 2020 COVID-19 pandemic between “younger” and “older” centenarians? And if there is any difference, when did the largest difference appear, considering the exact date of birth of centenarians?

To answer these questions, we try to increase the temporal resolution of the mortality analysis by considering the probability of dying according to month of birth. Owing to the difference in the months’ length and to avoid any possible seasonal effect on mortality risk, the EMR was calculated using a moving average aggregated with a group of 12 successive months.

**Figure 2:**
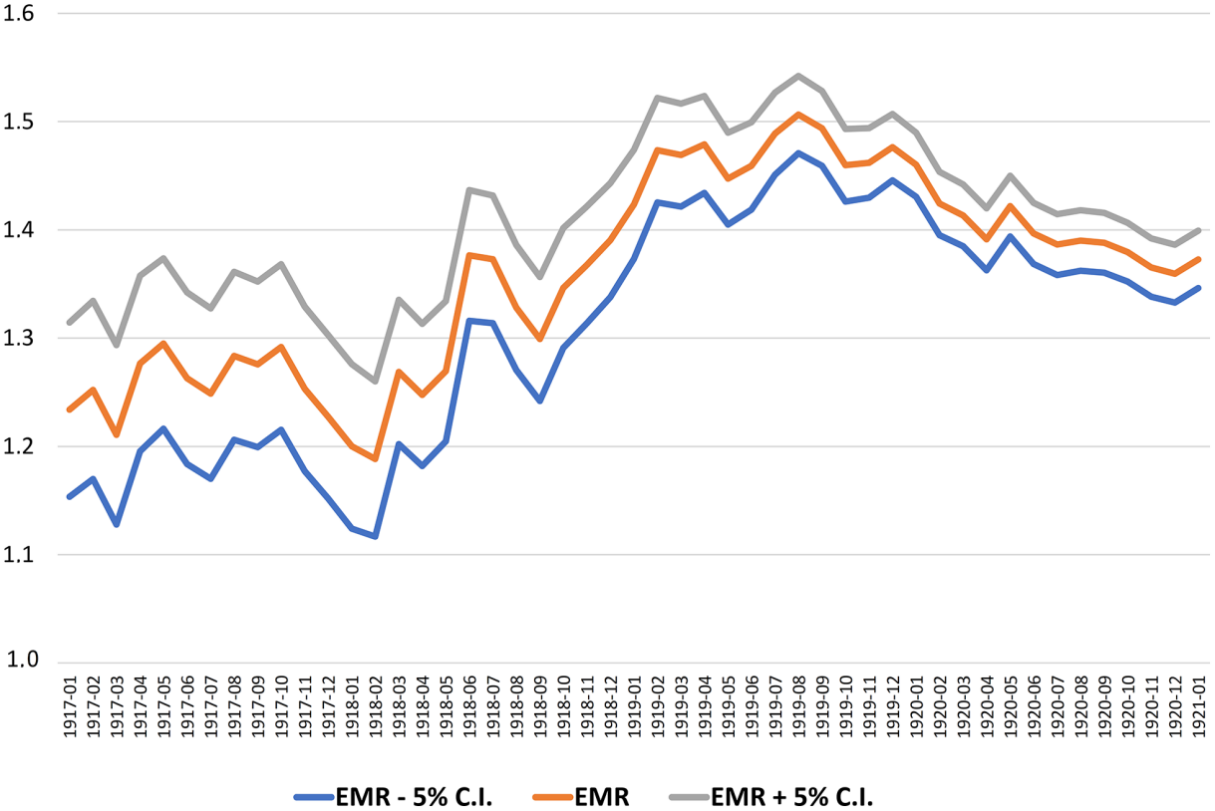
The EMR by month of birth with 5% confidence intervals (12 months moving average, 1917-1920).

Figure 2 illustrates the calculation of the EMR during the 2020 pandemic for those born in the years 1916-1921 by year and month of birth. Therefore, we use a 12-months moving average with 5% confidence intervals.

The greatest increase in the EMR corresponds to cohorts born between March 1918 and March 1919. To determine the point of maximum increase of EMR more accurately, we adopt a complementary method that compares the calculated EMR, as moving average, in two adjacent periods of 12 months each. The best cut point is defined as that which maximizes the difference between the two periods of 12 months, before and after that date.

**Figure 3:**
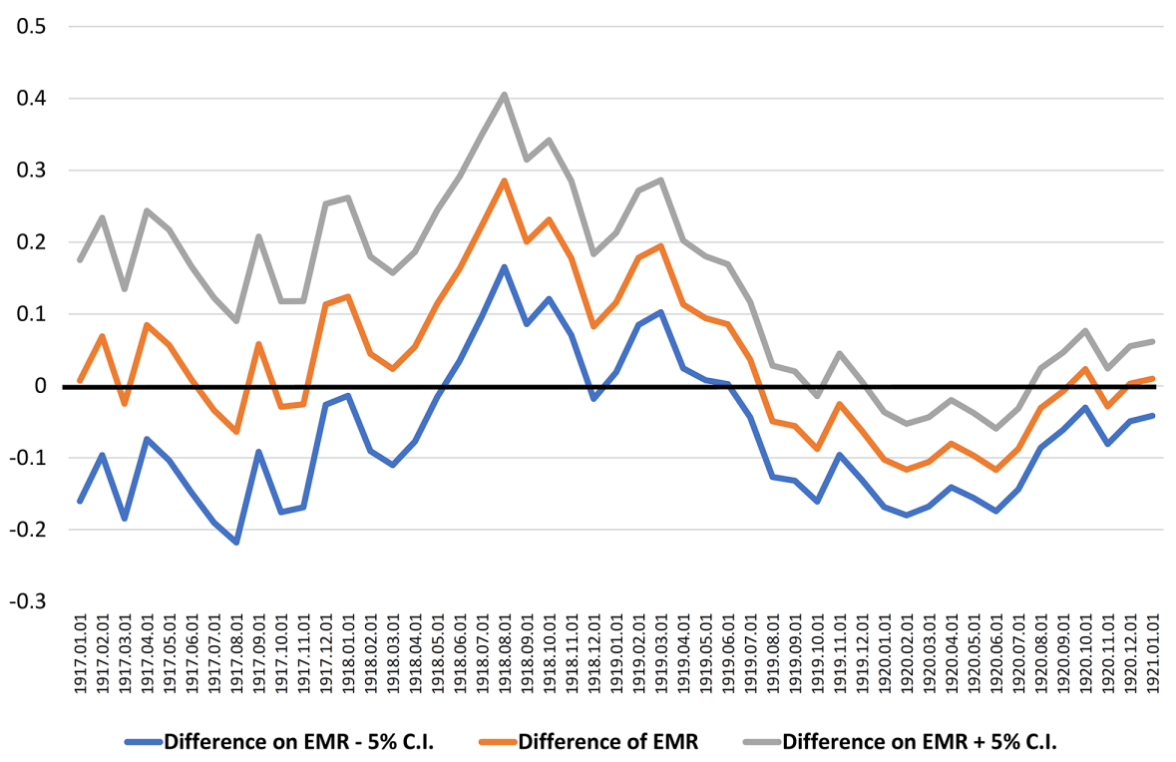
Identifying the best cut point as the larger difference of the EMR calculated between two adjacent periods of 12 months, from 1917-1920.

In Figure 3, the confidence intervals indicate that the difference is significantly different from zero during the aforementioned period, and the best cut point maximizing that difference coincides with August 1, 1918 with a higher 29% EMR for centenarians born in the 12 months after that date (EMR = 1.474) compared with those born in the 12 months before (EMR = 1.188).

## DISCUSSION

In this paper, using statistics on the oldest old population living in Belgium, we report that centenarians born before August 1, 1918, globally display a lower EMR during the 2020 COVID-19 pandemic, compared with centenarians born later. The difference is statistically significant at a level of 5%. Such a relative survival advantage among the “older” centenarians is unexpected and intriguing enough to be worthy of further investigations.

Two main events, during the second half of 1918, may be associated to the EMR difference during the 2020 pandemic as reported in this article: the end of WWI on November 11, 1918 and the outbreak of the Spanish flu due to H1N1 influenza virus, which was attested in Belgium since July 1918.

### The impact of the end of WWI

**Figure 4:**
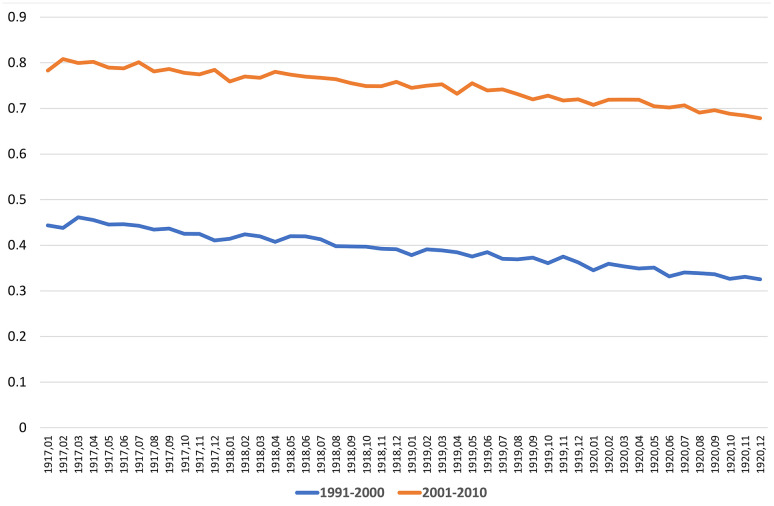
Probability of dying from 1991 until 2000 and from 2001 to 2010, calculated by year and month of births (STATBEL and data from Centre de Démographie, UCLouvain, Belgium).

WWI (1914-1918) led to a period of deprivation for the Belgian population suffering under German occupation. Especially in 1917 and 1918, the nutritional status and sanitary conditions of the population had further worsened until 1920 [10]. The impact of nutrient deficit and low-quality foods during wartime may have had devastating effects on both the short- and long-term health of the new-born. Protein-energy malnutrition may heavily affect the course of pregnancies, causing increased stillbirth rates [11]. Unfortunately, no data on the number of stillborn and only little data on the level of infant mortality during WWI are available in Belgium [12]. An increase in infant mortality was observed in 1917 and 1918, a similar trend also reported in the neighboring countries, e.g., France and the Netherlands [13]. Such an increase may have resulted in a stronger selection against the weakest babies, born in this period, considering that harsh privations persisted more than a year after the end of the war. Research on French children born during WWI revealed that, in general, parental socioeconomic conditions were a strong predictor of new-borns’ longevity [14]. Children whose mothers had faced famine during pregnancy tended to have a shorter lifespan [15]. These long-term effects have been ascribed to a direct fetal distress or mediated by epigenetic mechanisms [16]. Nevertheless, it is important to mention the strong support by the U.S. Commission for Relief in Belgium that improved the food supply for babies, thereby ensuring them better early-life conditions despite the poor socio-economic context lasting until the end of 1919 [17]. Although some kind of selection early in the life of these generations can hardly be excluded, its impact on their survival into old ages is unknown, and there is no consensus on the existence of its favorable or detrimental significance [18]. If such a selection was real and supposed to be favorable for survival, babies born during WWI or immediately after would be expected to have different mortality risks along their whole lifespan and not exclusively at the extreme end of life. To test the selection hypothesis, we disaggregated the mortality risks of babies born before and after the end of WWI, considering their month and year of births for the periods 1991-2000 and 2001-2010. The corresponding curves presented in Figure 4 do not show any evidence of a significant variation of mortality for people born around the end of WWI. We cogently conclude that a selection in terms of WWI can hardly be responsible for the relative better survival of ‘older’ centenarians compared with ‘younger’ ones against 2020 COVID-19, despite both groups likely experiencing similarly threatening early-life conditions during the three years around the end of WWI.

### The impact of “Spanish flu”

The second important event that may potentially explain our results is the outbreak of the Spanish flu that caused about 50,000 deaths by the fall of 1918, i.e., more than the estimated 44,000 victims of WWI [19]. As explained by Brulard in his dissertation [20], the Belgian press covering the timeline of the Spanish flu mentioned the latter for the first time on July 7, 1918. The overall death rate in the city of Brussels increased: from July 1 to July 20, 68 civilians of the age 10–40 years died, while the number of deaths for the same age group in June was 35. In the first half of August, the whole country was facing the virus but, very quickly, the pandemic subsided, only to re-emerge in October. This second wave was by far the most devastating as more than half of the victims attributed to the Spanish flu died between mid-October and mid-November. Thereafter, it gradually lessened in intensity, although many deaths linked to the Spanish flu were still recorded in December. A third wave resurfaced in January 1919, culminating in February. Although it was less deadly than the second wave, it testified to the persistent circulation of the virus, which did not disappear until the end of May 1919.

In our analysis, we detect a synchronic association between the timing of the Spanish flu pandemic and the switch in centenarians’ EMR. To better outline this potential association, we group the number of observed and expected deaths of centenarians during the 2020 pandemic into three periods of birth, namely, before, during and after the Spanish flu pandemic (Table 1). A significant difference in the EMR of centenarians in 2020 is evident between the “young” centenarians born after August 1, 1918 and the “older” ones born before that date.

**Table 1:** Number of expected and observed deaths of centenarians during the 2020 pandemic (March 10 - December 31) considering three periods, before, during and after the Spanish flu pandemic and estimated over-mortality.

**Period of observation**		**At risk population March 10, 1918**	**Observed deaths**	**Expected deaths**	**Excess mortality ratio**	**5% confidence interval**
January 1, 1917	July 31, 1918	501	180	144	125.0%	7.0%
August 1, 1918	May 31, 1919	370	138	96	143.8%	8.1%
June 1, 1919	December 31, 1920	2264	765	538	142.2%	3.4%

The near-perfect synchronism between the EMR gap during the 2020 pandemic for cohorts born before and after August 1, 1918 and the surge of the influenza pandemic naturally suggests a causal effect, which becomes plausible as we excluded the main alternative explanation based on the impact of WWI ending, especially considering the persistence of poor living conditions after the conflict.

For the exposure to the Spanish flu pandemic, three groups of newborns could be distinguished. The first group included babies who were already born before the pandemic broke out, and who faced the virus in their early life. The youngest among them could have been protected by maternal antibodies transferred through breastmilk during the first months of their life. Nonetheless, as the duration of the pandemic exceeded that of maternal protection, the majority of them had to face the virus. In contrast, babies born after August 1, 1918 but before the pandemic completely disappeared by the end of May 1919, could have been protected by maternal immunity, with a variable risk of coming in contact with the virus. Babies born after May 1919 might have been exposed to the H1N1 virus but only in utero and, if they survived, were protected by maternal immunity in their first months of life, while those born after January 1920 were not exposed to the virus at all.

Only the first group faced the virus expressing a significant survival advantage later in life during the 2020 COVID-19 pandemic. Our speculative hypothesis is that most of them could have developed immune memory cells capable of recognizing epitopes antigenically related to the H1N1 virus potentially even a century later. This hypothesis is supported by some scientific evidence. For instance, individuals born before 1957 and exposed to the H1N1 influenza A virus were better protected from the 2009 pH1N1 [21]. Even though the influenza and SARS-CoV-2 viruses are different, some structural homology between them has been reported [22] and a subset of the T cell repertoire capable of cross-reacting with both influenza virus and SARS-CoV-2 virus epitopes has been identified [23]. More specifically, SARS-CoV-2 T-cell repertoires with specificity to both the coronavirus and the M1 immunodominant epitope of influenza virus are more frequent that expected, which may have relevant implications in the response to COVID-19 for those individuals previously exposed to some influenza strains. Indeed, this raises the possibility that cohorts exposed to the Spanish flu in 1918 were capable to mount an effective response also against COVID-19 in 2020. Efficient memory cells can persist for many decades, as demonstrated by the study of Yu et al. [24] wherein individuals exposed to the H1N1 pandemic in 1918/1919 were still able to produce neutralizing antibodies a century later, confirming that a centenarian's immune system can successfully react to pathogens to which they were exposed early in life. However, this hypothesis is in contrast with that of Gagnon et al. [25] that exposure to the influenza pandemic early in life is a risk factor for dying during subsequent heterosubtypic pandemics. For this reason, a putative influenza / coronavirus cross-response should be viewed an interesting research hypothesis that will require further investigations aimed at better clarifying the underlying molecular mechanisms acting in the host.

### Strengths, limitations, and future research

The main strength of the presented study is the direct link established between the two greatest pandemics of the past one hundred years. Our investigations are innovative in that we study the only persons exposed to both pandemics, i.e., today’s centenarians. Fortunately, reliable and exhaustive data on centenarians and their survival is available in Belgium, and the sufficient number of people involved in our analysis allow a high level of statistical significance. Data concerning people born at the time of the Spanish flu and are still alive at the onset of the 2020 COVID-19 pandemic on March 10 are examined in great detail (according to month of birth), something that hardly occurs in such kind of research, taking into account the size of each birth cohort. Nevertheless, the study has several limitations. First, due to the small number of male centenarians, a separate analysis of males and females could not be performed. Another limitation is linked to the difficulty in distinguishing COVID-19-related deaths and those due to other causes. Further, only the overall mortality, including that due to COVID-19, is addressed as during the early stage of the pandemic, some cases are probably not attributed to COVID-19. Third, it cannot be ruled out that the susceptibility to COVID-19 among the oldest old is strongly influenced by the specific living conditions of this age group, as most centenarians were confined to nursing homes. Further research is on-going to assess the impact of these collective living arrangements on mortality during the pandemic. Finally, we do not carry out direct blood testing in the oldest people experiencing COVID-19, therefore our hypothesis of protection provided by a cross-reactive immunity elicited during a previous H1N1 pandemic remains entirely speculative. Nevertheless, the possibility that those exposed to the Spanish flu have developed immune mechanisms capable of inducing a more effective anti-COVID-19 response than non-exposed people born after it is intriguing and deserves further investigation across different populations.

## Supplementary Material

Supplementary Table 1
