# Supplementary material for: Centenarians exposed to the Spanish flu in their early life better survived to COVID-19
Source: Aging (Albany NY). 2021 Sep 27;13(18):21855–65. doi: 10.18632/aging.203577 (PMC8507269; doi:10.18632/aging.203577)
Supplement: Supplementary Table 1 [file aging-13-203577-s001.pdf]

SUPPLEMENTARY TABLE

Supplementary Table 1. Population alive as of March 10, 2020 by year and month of birth, observed and expected number of deaths between March 10 and December 31.

Year	Month	Age as of March 10, 2020	Number of persons alive	Observed deaths	Observed mortality rate	Expected mortality rate	Expected deaths
1916	January	104 years 2 months	3	2	66,7%	31,4%	1
1916	February	104 years 1 month	10	4	40,0%	31,2%	3
1916	March	104 years	8	6	75,0%	31,1%	2
1916	April	103 years 11 months	16	7	43,8%	30,9%	5
1916	May	103 years 10 months	12	6	50,0%	30,8%	4
1916	June	103 years 9 months	10	3	30,0%	30,6%	3
1916	July	103 years 8 months	17	6	35,3%	30,4%	5
1916	August	103 years 7 months	13	3	23,1%	30,3%	4
1916	September	103 years 6 months	16	5	31,3%	30,1%	5
1916	October	103 years 5 months	9	2	22,2%	30,0%	3
1916	November	103 years 4 months	15	5	33,3%	29,8%	4
1916	December	103 years 3 months	14	5	35,7%	29,7%	4
1917	January	103 years 2 months	12	4	33,3%	29,5%	4
1917	February	103 years 1 month	16	10	62,5%	29,3%	5
1917	March	103 years	24	7	29,2%	29,2%	7
1917	April	102 years 11 months	10	4	40,0%	29,0%	3
1917	May	102 years 10 months	21	10	47,6%	28,9%	6
1917	June	102 years 9 months	14	5	35,7%	28,7%	4
1917	July	102 years 8 months	7	3	42,9%	28,6%	2
1917	August	102 years 7 months	17	2	11,8%	28,4%	5
1917	September	102 years 6 months	16	8	50,0%	28,3%	5
1917	October	102 years 5 months	18	6	33,3%	28,1%	5
1917	November	102 years 4 months	18	4	22,2%	28,0%	5
1917	December	102 years 3 months	20	6	30,0%	27,8%	6
1918	January	102 years 2 months	13	6	46,2%	27,7%	4
1918	February	102 years 1 month	24	12	50,0%	27,5%	7
1918	March	102 years	23	7	30,4%	27,4%	6
1918	April	101 years 11 months	20	5	25,0%	27,2%	5
1918	May	101 years 10 months	30	11	36,7%	27,1%	8
1918	June	101 years 9 months	17	4	23,5%	26,9%	5
1918	July	101 years 8 months	38	12	31,6%	26,8%	10
1918	August	101 years 7 months	40	15	37,5%	26,6%	11
1918	September	101 years 6 months	31	11	35,5%	26,5%	8
1918	October	101 years 5 months	23	9	39,1%	26,3%	6
1918	November	101 years 4 months	37	19	51,4%	26,2%	10
1918	December	101 years 3 months	39	12	30,8%	26,0%	10
1919	January	101 years 2 months	40	11	27,5%	25,9%	10
1919	February	101 years 1 month	40	14	35,0%	25,8%	10
1919	March	101 years	37	16	43,2%	25,6%	9
1919	April	100 years 11 months	38	13	34,2%	25,5%	10
1919	May	100 years 10 months	45	18	40,0%	25,3%	11
1919	June	100 years 9 months	47	18	38,3%	25,2%	12
1919	July	100 years 8 months	68	28	41,2%	25,0%	17
1919	August	100 years 7 months	58	20	34,5%	24,9%	14
1919	September	100 years 6 months	98	36	36,7%	24,8%	24
1919	October	100 years 5 months	102	32	31,4%	24,6%	25
1919	November	100 years 4 months	107	45	42,1%	24,5%	26
1919	December	100 years 3 months	118	45	38,1%	24,3%	29

1920	January	100 years 2 months	143	51	35,7%	24,2%	35
1920	February	100 years 1 month	112	36	32,1%	24,1%	27
1920	March	100 years	126	38	30,2%	23,9%	30
1920	April	99 years 11 months	134	46	34,3%	23,8%	32
1920	May	99 years 10 months	154	59	38,3%	23,6%	36
1920	June	99 years 9 months	161	51	31,7%	23,5%	38
1920	July	99 years 8 months	145	40	27,6%	23,4%	34
1920	August	99 years 7 months	153	46	30,1%	23,2%	36
1920	September	99 years 6 months	112	30	26,8%	23,1%	26
1920	October	99 years 5 months	151	57	37,7%	23,0%	35
1920	November	99 years 4 months	123	38	30,9%	22,8%	28
1920	December	99 years 3 months	152	49	32,2%	22,7%	34
1921	January	99 years 2 months	168	57	33,9%	22,5%	38
1921	February	99 years 1 month	168	50	29,8%	22,4%	38
1921	March	99 years	199	54	27,1%	22,2%	44
1921	April	98 years 11 months	197	56	28,4%	22,1%	44
1921	May	98 years 10 months	194	65	33,5%	22,0%	43
1921	June	98 years 9 months	191	62	32,5%	21,8%	42
1921	July	98 years 8 months	222	69	31,1%	21,7%	48
1921	August	98 years 7 months	239	74	31,0%	21,5%	51
1921	September	98 years 6 months	230	67	29,1%	21,4%	49
1921	October	98 years 5 months	232	67	28,9%	21,2%	49
1921	November	98 years 4 months	234	71	30,3%	21,1%	49
1921	December	98 years 3 months	280	81	28,9%	21,0%	59
